# Potential for chromium (VI) bioremediation by the aquatic carnivorous plant *Utricularia gibba* L. (Lentibulariaceae)

**DOI:** 10.1007/s11356-015-4151-1

**Published:** 2015-01-31

**Authors:** Joanna Augustynowicz, Krzysztof Łukowicz, Krzysztof Tokarz, Bartosz Jan Płachno

**Affiliations:** 1 Unit of Botany and Plant Physiology, Institute of Plant Biology and Biotechnology, Faculty of Biotechnology and Horticulture, University of Agriculture in Kraków, Al. 29 Listopada 54, 31-425 Kraków, Poland; 2 Faculty of Animal Science, University of Agriculture in Kraków, Al. Mickiewicza 24, 30-059 Kraków, Poland; 3 Department of Plant Cytology and Embryology, Jagiellonian University in Kraków, Gronostajowa 9 St., 30-387 Kraków, Poland

**Keywords:** Aquatic carnivorous plants, Chlorophyll fluorescence, Chromium, Heavy metals, Phytoremediation

## Abstract

The aquatic carnivorous plant *Utricularia gibba* has one of the smallest known genomes among flowering plants, and therefore, it is an excellent model organism for physiological and developmental studies. The main aim of our work was to check whether the ubiquitous *U. gibba* might be useful for the phytoremediation of the highly toxic and mobile hexavalent chromium in waters. Plants were incubated for 1 week in a 50 μM (2.6 mg dm^−3^) Cr(VI) solution in laboratory conditions. Our results revealed that the plant exhibits a very high accumulation capacity for Cr. The accumulation level was higher than 780 mg kg^−1^ and a bioconcentration factor >300. On the other hand, the plants showed a low tolerance to the elevated Cr concentration, which was expressed in a significant decrease of the photosystem II activity. However, the most pronounced negative influence of chromate was found on the morphology and activity of the traps. Due to its high accumulation capacity, we suggest that *U. gibba* may be efficient in the removal of chromate over a short time scale. It can also provide a new molecular resource for studying the mechanisms of Cr(VI) detoxification.

## Introduction

The Water Framework Directive (WFD) has been in force across the member countries of the European Union since 2000. The main objective of the WFD is to ensure the protection of water and suitable levels of water ecology and chemical purity. In cleaning up the environment, there is an alternative to the physicochemical methods in the form of biological methods. Phytoremediation particularly denotes the use of plants to remove pollutants from water, soil, and air and/or to transform these pollutants into less harmful forms. Plants are capable of absorbing metal pollutants and transferring them to their aboveground parts (phytoextraction) and—as is the case for some species—of accumulating them in large quantities (phytoaccumulation) (Ali et al. 2013).

Chromium occurs naturally in two stable oxidation forms, which are Cr(III) and Cr(VI). However, these two forms are principally different in their physicochemical properties and toxicities. In solutions, Cr(VI) occurs as a chromate or dichromate anion. It is an extremely strong oxidant that is highly soluble within a wide range of pH and thus is easily bioavailable (Kotaś and Stasicka 2000). It can act as an oxidizing agent itself, and it can also induce the formation of free radicals during its conversion to Cr(III). Hexavalent chromium has mutagenic and carcinogenic effects on humans and animals. It is also highly toxic to plants (Saha et al. 2011; Zayed and Terry 2003). Cr(III) is less toxic, and in low concentrations, it is a microelement in the diet of mammals (Schwartz and Mertz 1959). Chromium pollution (resulting mainly from metal industries and leather manufacturers) constitutes a significant issue not only in developed countries (e.g., the USA) but also in developing ones (e.g., India). Because of the concentrations of chromium in waters, which often exceed the permissible levels, both Cr(VI) and Cr(III) compounds are regarded as priority toxic pollutants (EPA Water Quality Standards) by the US Environmental Protection Agency (US EPA).

The phytoextraction/phytoaccumulation of metals in natural water ecosystems, which is based on the use of aquatic vascular plants (macrophytes), is a competitive strategy compared to other bioremediation methods. The benefits of using vascular plants include eliminating pollutants in in situ conditions, the easy collection of biological material, and the absence of risk to the migration of organisms in soil-aquatic environments. Several macrohydrophytes and wetland plants are used for the bioremediation of water that has been polluted by metallic compounds (e.g., Augustynowicz et al. 2010, 2014; Axtell et al. 2003; Deng et al. 2004; Malec et al. 2011; Ye et al. 1997a, b).

*Utricularia gibba* is a ubiquitous carnivorous plant. There are more than 800 species of carnivorous plants that trap, kill, and absorb nutrients from various organisms (Darnowski et al. 2006; Juniper et al. 1989; Król et al. 2012). Some of these (*Sarracenia purpurea* ssp. *purpurea*, *Drosera rotundifolia*, *Pinguicula* sp.) were recorded in environments with soil that was rich in heavy metals (D’Alessi 2004). There have also been observations of two species of bladderworts (*Utricularia australis* and *Utricularia intermedia*) in aquatic environments in Poland that had been heavily polluted by Tl as well as by other heavy metals (Cd, Pb, and Zn) (Płachno and Augustynowicz unp.). Why did we choose *U. gibba*? This species, which was recently sequenced and analyzed (Ibarra-Laclette et al. 2011 and 2013), like *Genlisea margaretae* (63 Mb, Greilhuber et al. 2006) and *Genlisea aurea* (63.6 Mb, Leushkin et al. 2013), has one of the smallest known genomes in flowering plants (82-Mb genome—approximately half that of *Arabidopsis*). Thus, *U. gibba* is an excellent model organism for physiological and developmental studies; some of these have begun (e.g., Chormanski and Richards 2012; Juang et al. 2011). Moreover, it has a broad geographic range (all continents except for Antarctica and it was introduced into Europe) and plasticity in the case of its habitats (Taylor 1989). *U. gibba* is also small in size, and it is easy to cultivate (D’Amato 1998; Schnell 2002).

The main aims of our study were:

- to determine the ability of *U. gibba* to accumulate and tolerate heavy metals in its tissues,
- to assess the potential use of *U. gibba* in phytoremediation.

In order to implement the objectives, we tested the accumulation capacity of the species for Cr. The biochemical aspects of Cr(VI)’s influence on plant metabolism like the activity of photosystem II (PSII) as well as chlorophyll *a*, chlorophyll *b*, and carotenoid content were analyzed. We also performed a detailed analysis of the activity and morphology of the traps that were under the influence of Cr(VI).

## Material and methods

### Plant material and accumulation tests

Plants of *U. gibba* L. (subgenus *Utricularia*, section *Utricularia*) were obtained from the Botanical Garden of Jagiellonian University in Kraków, Poland, and later cultured in the Institute of Plant Biology and Biotechnology at the University of Agriculture in Kraków. The light conditions of the plant cultures and the composition of the medium were chosen based on the results of 2-month preliminary tests. Before starting the experiments with Cr, the shoots were rinsed several times with distilled water after tap water. A Cr solution was prepared using twice-diluted macroelements and microelements of a standard MS medium, pH 5.4, supplemented with 50 μM (2.6 mg dm^−3^) Cr(VI) (as K_2_CrO_4_; POCh Gliwice, Poland). This Cr concentration was selected based on the results of earlier experiments of the authors (Augustynowicz et al. 2010). Two grams of plant biomass were incubated in 200 cm^−3^ solutions. Incubations were conducted for 7 days under controlled conditions: 16-h photon flux density ca. 40 μmol m^−2^ s^−1^ and 8-h darkness, 23–24 °C. The control samples were plants that were incubated as described above but with no Cr added.

### Cr content analysis

Before the analysis of Cr content in the shoots, the plant material was thoroughly washed three times with distilled water and then dried for 24 h at 105 °C. Digestion was performed in a mixture of H_2_O_2_ and HNO_3_ (6:1; *v*/*v*) (Suprapur, Merck) in a closed system of a microwave oven (Multiwave 3000, AntonPaar). Inductively coupled plasma mass spectrometry (ICP-MS) (ELAN 6100, Perkin Elmer) was used to measure the amount of Cr in the samples. The spectrometer was calibrated using the ICP multielement standard (Merck).

### Measurements of physiological status of plants

Hydration of *Utricularia* shoots was measured after drying the plants at 105 °C for 24 h. Photosynthetic pigments, chlorophylls *a* and *b*, and carotenoids were isolated using the method described by Świderski (1998) in which 0.1 g of the shoots was homogenized in acetone (POCh Gliwice, Poland) with a small amount of CaCO_3_ (POCH Gliwice, Poland) in order to neutralize the organic acids, after which they were centrifuged for 15 min, 5400 g, at 4 °C (Rotina 380-R, Hettich Zentrifugen, Germany). The pigment content was calculated in accordance with the equations presented by Lichtenthaler and Wellburn (1983) after the measurements of absorbance (UV-Vis spectrophotometer, HITACHI U-2900) at 470, 645, and 662 nm.

The chlorophyll fluorescence was determined using a chlorophyll fluorescence monitoring system (FluorCam, Photon Systems Instruments, Czech Republic) according to the standard procedure as modified for water plants as described by Augustynowicz et al. (2014). The PSII maximal photochemical quantum yield (F_V_/F_M_), which was calculated as (F_M_ − F_0_) / F_M_, was obtained after dark adaptation for at least 20 min, which is necessary for the measurement of the basic chlorophyll fluorescence yield (*F*_0_). The maximal chlorophyll fluorescence yield (F_M_) was recorded by applying a 0.8-s saturating light pulse (2000 μmol m^−2^ s^−1^).

An original *Paramecium* test was developed in order to detect the activity of the traps that were under the influence of Cr. *Paramecia* were house-stock cultured in 500 ml glass containers at room temperature and stored out of direct sunlight for 7 days. The house-stock was prepared using dechlorinated tap water containing 3–5 g of hay and banana peel. The composition of the hay was as follows: *Plantago maior*, *Plantago lanceolata*, *Taraxacum officinale*, *Urtica dioica*, and *Achillea millefolium*. It was commercially available hay for small rodents. The culture was regularly aerated. Before application, the vitality and number of *Paramecium* cells were analyzed. In order to measure the activity of the traps, 1 cm^−3^ of the described *Paramecium* stock was incubated with 0.5 g of *Utricularia* in a 25 cm^−3^ culture medium (Cr-containing or control) for 48 h. After the incubation, shoots of *Utricularia* were exhaustively washed with a half-strength MS medium to detach any protozoa cells that may have remained on the surface. The activity of the traps was expressed as the number of *Paramecium* that had been absorbed by the traps. It was computed as the difference between the number of *Paramecium* in the incubation medium at the start and at the end of the incubation. The quantity of *Paramecia* was calculated under an optical microscope (Opta-Tech, Poland) using a Bürker chamber. For that reason, the protozoa were treated for 2 min at −20 °C in order to immobilize them. The total volume of solutions used and the fresh weight of shoots were always taken into account in the calculations. The negative effect of Cr(VI) on *Paramecium* vitality was also tested. No influence of Cr(VI) on the number or mobility of *Paramecium* was found under the conditions of the described test.

### Microscopy analysis

#### Light microscopy

Fresh plant material was examined using an Olympus BX60 fluorescence/light microscope.

#### Scanning electron microscopy

Material was fixed in 5 % glutaraldehyde in a phosphate buffer (pH 7.4). The samples were dehydrated in serial dilutions of ethanol and acetone, after which they were critical point dried in liquid CO_2_ and coated with gold using a JEOL-JFC 1100E sputter coater. The specimens were viewed under a HITACHI S-4700 microscope (Scanning Microscopy Laboratory of Biological and Geological Sciences, Jagiellonian University in Kraków) at 20 kV.

### Statistics

The Student’s *t* test or Mann-Whitney *U* test (if data were not normally distributed) were used to compare any significant differences in pairs (control and Cr-treated samples) at *α* = 0.05 based on STATISTICA ver. 10 software (StatSoft Inc. 2011). Up to four sets of independent experiments with several independent replicates in each set were performed.

## Results

Cr accumulation tests revealed that after a 7-day incubation in a chromate solution, the shoots of *Utricularia* were able to accumulate an average of 787.4 mg kg^−1^ (±179.6) (*n* = 4). Cr in the control plants was detected at an average level of 3.3 mg kg^−1^ (±2.1) (*n* = 4). The bioconcentration factor (BCF), which is defined as the ratio of Cr quantity in tissue (mg kg^−1^ d.w.) to the Cr quantity in solution (mg kg^−1^), was slightly above 300.

**Fig. 1:**
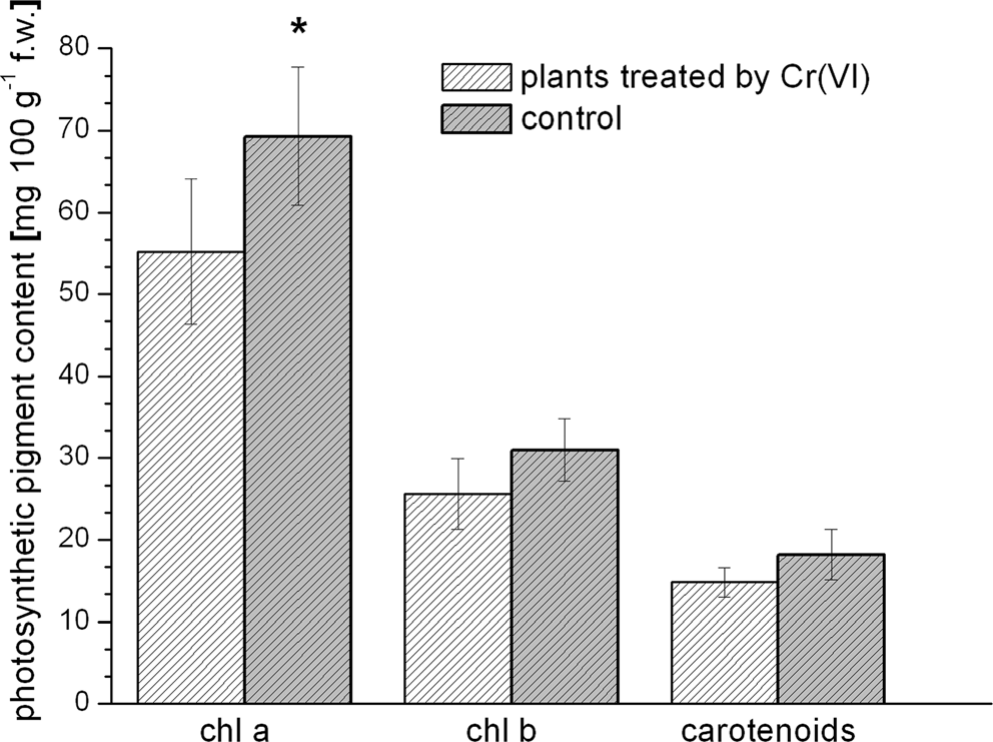
The content (mg g^−1^ fresh weight) of chlorophyll *a*, chlorophyll *b*, and carotenoids in the control as well as in the Cr(VI)-treated plants. Statistically significant differences in pairs were found for chl *a*, which are marked with an *asterisk* (Student’s *t* test at *α* = 0.05; chl *a p* < 0.023; chl *b p* < 0.065; carotenoids *p* < 0.074). *Error bars* represent SDs; *n* = 6

**Fig. 2:**
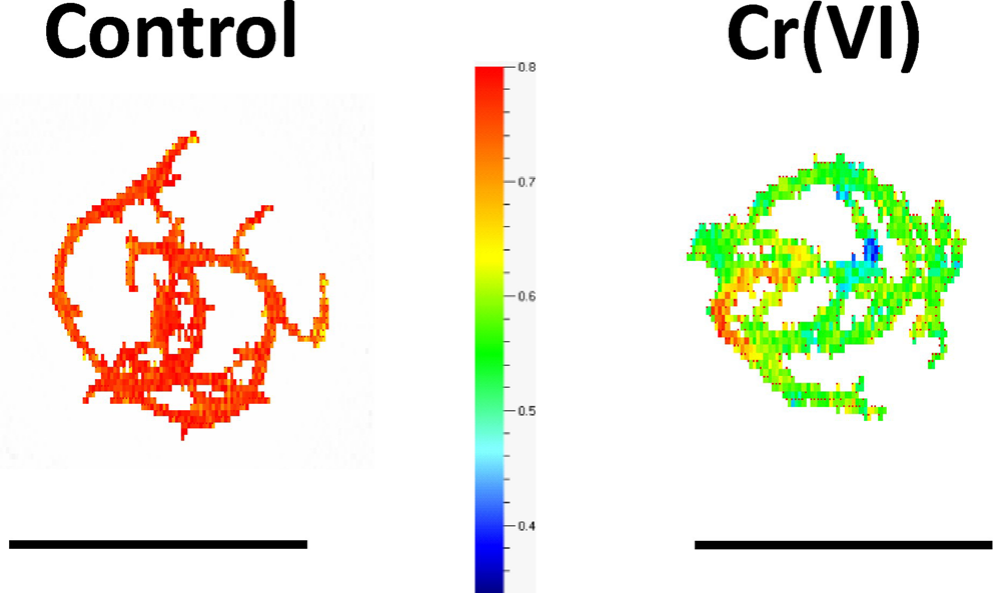
Representative spatial distribution of the fluorescence emission of bladderwort shoots. The *color scale* represents the PSII maximal photochemical quantum yield (F_V_/F_M_). *Bar* = 30 mm

Regarding the physiological status of shoots, we did not observe any differences in the tissue hydration between the Cr-influenced and control plants. The dried mass accounted for 7.82 % (±0.75) or 7.74 % (±1.34) in the case of the Cr-influenced and control plants, respectively (*p* > 0.05; *n* = 7). There were also no differences in the general morphology of the shoots between the control and Cr(VI)-exposed plants. On the other hand, a decrease in the content of photosynthetic pigments was detected, although Cr(VI) did not severely affect the concentration of photosynthetic pigments after the 7-day incubation. Significant statistical differences between the control and Cr-exposed plants were observed only for chlorophyll *a.* The level of chlorophyll *b* and carotenoids were lower in the stressed plants but not significantly (Fig. 1). The impact of hexavalent chromium on a plant’s photosynthetic apparatus was also measured based on the analysis of chlorophyll *a* fluorescence. It was manifested by a statistically significant decrease in the PSII maximal photochemical quantum yield (F_V_/F_M_). The spatial distribution of the F_V_/F_M_ parameter in *Utricularia* shoots is presented in Fig. 2. The lower PSII efficiency was related to a statistically significant increase in the basic chlorophyll fluorescence yield (F_0_) in the bladderwort shoots that had been incubated in the chromate solution, which is in accordance with the decrease in chlorophyll *a*. In contrast, the variable fluorescence (F_V_) stayed at a level that was similar to the control plants (Table 1).

**Table 1:** Cr(VI) impact on selected fluorescence parameters of bladderworts shoots

	Control	Cr-influenced	Significance
F_0_	80.0 ± 19.0	135.7 ± 16.5	*
F_V_	319.3 ± 88.2	336.8 ± 28.2	ns
F_V_/F_M_	0.7 ± 0.0	0.6 ± 0.1	*

Asterisk indicates significant difference (Student *t* test at *α* = 0.05; F_0_ *p* < 0.009; F_V_ *p* < 0.300; F_V_/F_M_ *p* < 0.001

**Fig. 3:**
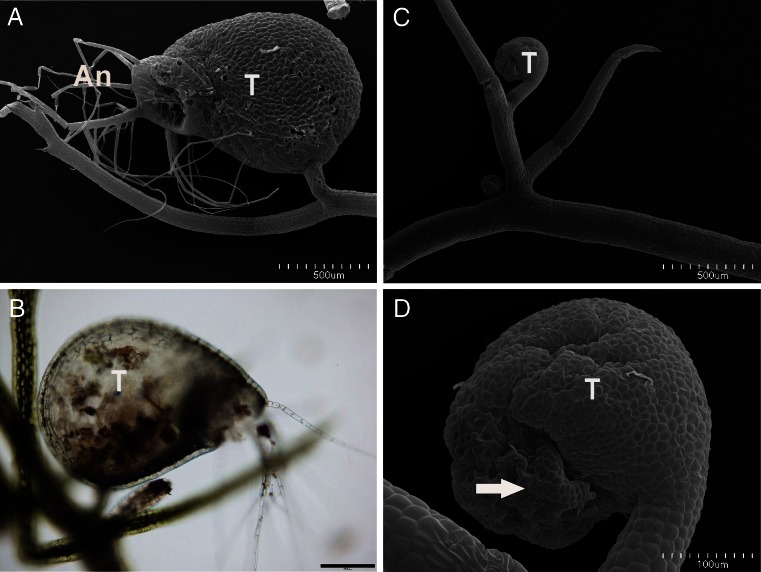
Morphology of traps of *Utricularia gibba*. **a**, **b** Traps of the control plants, *bars* = 500 and 200 μm. **c**, **d** Traps of the Cr(VI)-treated plants, *bars* = 500 and 100 μm. *T* trap, *A* antennae; reduced antennae (*arrow*)

The results that were obtained showed pronounced changes in the activity as well as in the morphology of the traps that had been subjected to Cr(VI). The average number of *Paramecia* that were absorbed by the Cr(VI)-treated traps was approximately three times lower than in the case of the control samples (Table 2). Although the activity of the traps was disturbed, the number of traps on shoots did not change under the influence of chromate even after the prolonged time of the experiment (data not shown). The altered activity of the traps, however, correlated with the morphological distortion of traps. In comparison to the control (Fig. 3a, b), the new traps of the plants that had been subjected to Cr(VI) were inhibited in development and were not functional (Fig. 3c, d). These immature traps did not have a functional trap door with trigger hairs, and therefore, they could not catch *Paramecia* (Fig. 3d). Plants also produced these nonfunctional traps after the prolonged time of the experiment (data not shown).

**Table 2:** Activity of traps expressed as a number of *Paramecium* adsorbed b*y* traps (of 1 g f.w.) during 48-h incubation of *Utricularia* plants in Cr(VI)-containing and control medium

	Mean	SD	Min	Max
Control	2.93 × 10^7^	0.85 × 10^7^	2.16 × 10^7^	4.10 × 10^7^
Cr-influenced	1.07 × 10^7^	0.82 × 10^7^	0.29 × 10^7^	2.12 × 10^7^

Significant differences were found between groups according to nonparametric Mann-Whitney *U* test (α = 0.05; *p* < 0.030). Two sets of independent experiments with two independent replicates in each set and 20 independent counts in each replicate were performed

## Discussion

The average chromium content in surface waters is usually at a low level, typically between a few fractions of microgram to a few micrograms per cubic decimeter (Kabata-Pendias and Mukherjee 2007). The quantity of Cr(VI) in surface waters that is acceptable by the US EPA (EPA Water Quality Standards) is 15 μg dm^−3^. With respect to this level, the concentration used in our experiment (50 μM = 2.6 mg dm^−3^) was more than 170 times higher and corresponded to the Cr amount in a polluted river environment (e.g., Liu et al. 2013). *U. gibba* was able to effectively accumulate this toxic element in such heavily Cr(VI)-polluted water. However, it must be stressed that a comparison between different plant species, which is related to the phytoextraction potential of a particular element, is sometimes difficult to perform. This is due to the fact that it depends on a number of factors: the time of the incubation, the concentration of metallic compound, the chemical form of the metal that is used, pH, the redox potential of the medium etc. Nevertheless, we showed that the accumulation level of Cr by *U. gibba* was more than a magnitude higher when compared to other free-floating macrophytes such as *Pistia stratiotes*, *Spirodela polyrhiza*, and *Eichhornia crassipes* (Mishra and Tripathi 2008), which grow in similar Cr(VI)-containing hydroponic cultures. These plants, especially *E. crassipess*, are known to be extraordinary phytoremediators of heavy metallic compounds in aquatic systems (Chandra and Kulshreshtha 2004; Zayed and Terry 2003; Sundaramoorthy et al. 2010). The abovementioned macrophytes when growing in a Cr(VI) concentration analogous to the one used in our experiment accumulated 306 < 98 < 71 (mg kg^−1^ d.w.) for *E. crassipes* < *P. startiotes* < *S. polyrhiza*, respectively (Mishra and Tripathi 2008). In another work, Giri and Patel (2011) showed that *E. crassipes* that had been grown in a hydroponic culture that was supplemented with 2.5 mg dm^−3^ of Cr(VI) for 6 days was able to accumulate ca. 800 mg kg^−1^ d.w. of chromium, which is a value similar to that of the *U. gibba* that is presented in our work. Moreover, the accumulation of Cr by bladderwort was comparable to that of an outstanding Cr phytoremediator—a submersed *Callitriche cophocarpa* that had been cultured in 50 μM Cr(VI) under similar laboratory conditions (Augustynowicz et al. 2010). We also showed that *U. gibba* exhibited a high BCF = 300.

In our opinion, the uptake of Cr is performed by the whole plant surface. However, it should be emphasized that the ratio of the weight of the traps and the shoots is very low. Therefore, we believe that the plant’s shoots are mainly responsible for the high Cr accumulation. Moreover, based on the results that are related to the Cr uptake, we would conclude that bladderwort can be considered to be a species that has a possible application for Cr(VI) phytoextraction purposes in aquatic systems. Unfortunately, an estimate of the accumulation of an element and its BCF is not enough to determine the application of a particular plant species in the cleanup of a polluted environment. The ideal plant for the practical use in phytoremediation, aside from exhibiting a considerable capacity for the metal uptake/degradation of xenobiotic, must also show i.a. a high tolerance for pollution. *U. gibba* was not found to be a plant that is highly resistant to Cr(VI). The 50 μM Cr(VI) that was used in our experiment induced a decrease in the photosynthetic pigment content in bladderwort tissues when compared to the control. Although the presence of chromate in the solution did not significantly alter the content of chlorophyll *b* and carotenoids, a statistically significant decrease in the quantity of chlorophyll *a* and fluorescence was observed. Our findings are in line with the literature data since a decrease in the photosynthetic pigment content is known to be the most sensitive response of plants to an increased heavy metal background (Appenroth 2010). *C. cophocarpa* that had been grown in the same Cr(VI) concentration for 5 days also showed some decrease in the pigment content, although the chlorophyll *a* fluorescence was not altered by Cr(VI) under these conditions (Augustynowicz et al. 2010). On the other hand, floating *P. stratiotes* that had been exposed to Cr-rich wastewater (35 and 50 mg dm^−3^) showed almost no influence of Cr on the chlorophyll content (Ganesh et al. 2008). The authors, however, did not distinguish the oxidation form of chromium that was used. The impact of Cr on living organisms depends very strongly on the form that is used. Hexavalent Cr compounds have been estimated to be one or even two magnitudes more toxic than trivalent compounds (Zayed and Terry 2003). *U. gibba* is a floating rootless plant, and therefore, its tolerance to pollution may also be lower than floating but rooted macrophytes like *E. crassipess* or *P. stratiotes*. These species accumulate most of the load of heavy metals in their root systems (Ganesh et al. 2008; Giri and Patel 2011), thus limiting the transfer of pollution to their mesophyll photosynthesizing tissues.

The impact of Cr on the photosynthetic apparatus may be related to either the donor or acceptor side of PSII reaction center (RC), PSI, as well as chloroplast ultrastructure disorganization (Shanker et al. 2005). In the case of *U. gibba*, the impact of Cr, which was manifested by a decrease in the chlorophyll *a* content as well as a high value of the basic chlorophyll fluorescence yield (F_0_), might have resulted from the ultrastructure of the degradation of the chloroplasts. It was previously reported that the degradation of the chlorophyll membrane leads to a decrease in the energy transfer rate from the light-harvesting chlorophyll (chl) *a*/*b* protein complexes to PSII (Domínguez et al. 2011). In addition, Cr is possibly responsible for the reduced size of the peripheral part of the antenna complex and may inactivate the activity of the enzymes that are involved in chlorophyll biosynthesis (Shanker et al. 2005). A high value of the basic chlorophyll fluorescence yield (F_0_) and a lower value of the maximal chlorophyll fluorescence (F_V_/F_M_) might reflect a permanent oxidation state of PSII reaction centers (Domínguez et al. 2011). Such changes might be caused by the oxidative activity of Cr, which additionally may be responsible for damage to the RC of PSII, which is in line with the observations of other authors (Shanker et al. 2005; Stefanov et al. 1995; Domínguez et al. 2011).

Despite the decrease of chl *a* content and the activity of PSII, the whole plant was able to carry out photosynthesis in the presence of Cr(VI). Furthermore, the hydration of the plant did not change despite the fact that changes in the water balance (in addition to changes in the content of chlorophylls) are considered to be the first symptoms of the negative impact of heavy metals on plants (Appenroth 2010). However, the most pronounced negative influence of Cr(VI) was on the morphology and activity of the traps. Aquatic rootless carnivorous *Utricularia* usually grow in nutrient-poor waters and because of this prey is an important source of nutrients (mainly nitrogen and phosphorous) to these plants. It was found that prey capture in aquatic *Utricularia* leads to more rapid growth (Adamec 1997; Adamec 2008). Aquatic *Utricularia* traps are inhabited by various commensals (e.g., Sirová et al. 2009, 2010; Płachno et al. 2012). The host plant even grows bacteria and ciliates. *Utricularia* supplies available organic C to the microbial community that thrives within the trap environment while benefiting from its by-products/nutrients (Sirová et al. 2010). Chromate severely influenced the activity and morphology of the traps. Thus, the plants lost an additional source of nitrogen and phosphorus from both the carnivory and commensal communities that thrive within the trap, which may cause a loss of competitiveness of *Utricularia* in comparison with other macrophytes.

## Conclusions

In our work, we proved that *U. gibba* can effectively extract Cr when it grows in water that has been polluted by the highly toxic Cr(VI) form. This element, however, negatively influenced the photosynthesis of the plant that was investigated, and it also influenced the activity and morphology of the traps far more severely. Nevertheless, due to its high Cr(VI) accumulation capacity, we suggest that *U. gibba* may be efficient in the removal of chromate over a short time scale, even if the plant’s metabolism is altered. *U. gibba* may be an especially good species for chrome removal in oligotrophic environments that have low water pH and poor macrophyte flora.
